# Programmed Death-1 Ligand 1 Domain Organization, Signaling Motifs, and Interactors in Cancer Immunotherapy

**DOI:** 10.3390/cancers17101635

**Published:** 2025-05-12

**Authors:** David Escors, Luisa Chocarro, Miriam Echaide, Claudia Rodriguez-Neira, Borja Vilaplana, Grazyna Kochan

**Affiliations:** OncoImmunology Unit, Navarrabiomed-Fundacion Miguel Servet, Hospital Universitario de Navarra (HUN), Instituto de Investigación Sanitaria de Navarra (IdISNA), Universidad Publica de Navarra (UPNA), 31008 Pamplona, Spainmiriam.echaide.gorriz@navarra.es (M.E.); claudia.rodriguez.neira@navarra.es (C.R.-N.); borja.vilaplana.marti@navarra.es (B.V.)

**Keywords:** cancer immunotherapy, immune checkpoint blockade, signal transduction pathways

## Abstract

Immunotherapies targeting the programmed cell death-1 ligand 1 (PD-L1) and programmed cell death 1 (PD-1) pathway sparked a revolution in cancer treatment. These breakthrough therapies work by disrupting the interaction between PD-1—expressed on T cells—and its ligand PD-L1, commonly found on the surface of cancer cells. By using monoclonal antibodies to block this binding, the immune system is unleashed to fight cancer more effectively. However, PD-L1’s role extends far beyond immune evasion. When situated on cancer cells, PD-L1 transmits inhibitory signals through PD-1, silencing the effector functions of T cells. However, PD-L1 also engages in reverse signaling, also called intrinsic signaling, delivering intracellular instructions that contribute to cancer cell survival, even in the absence of PD-1 binding. This signaling cascade shields cancer cells from apoptosis, drives proliferation, regulates DNA damage responses, and even functions as a co-transcriptional transactivator, amplifying cancer’s ability to thrive. The intricate mechanisms behind PD-L1’s intrinsic signaling are under intense investigation. In this review, we provide a historical perspective on the discoveries leading to PD-L1’s structure, signaling motifs, and interacting partners, shedding light on its multifaceted roles and the promising therapeutic possibilities ahead.

## Simple Summary

Immune checkpoint blockade therapies transformed clinical oncology. However, much is unknown of the cellular and immunological mechanisms behind its success. In recent years, extensive research on the mechanisms of action of programmed death-1 ligand 1 (PD-L1) uncovered the multiple facets of this immune checkpoint molecule on the biology of the cell. Here we review the current knowledge on PD-L1 structure, signaling motifs and interactors that provide a mechanistic insight into the functions of this key immune checkpoint.

## 1. Introduction

Not so long ago, cancer immunotherapies were not considered serious therapeutic options by most oncologists or researchers in oncology. Development of antineoplastic treatments was largely devoted to engineering small molecules and kinase inhibitors targeting cancer cell growth and survival pathways [1,2,3,4]. Cancer cells were the targets, and pharmaceutical companies invested significantly into research in cancer cell apoptosis, tumor angiogenesis, and processes around the cancer cell. However, focus was not centered in the patient’s immune system unless in collaboration with academic groups. These strategies were strongly incentivized by the emergence of targeted therapies with small molecule inhibitors specifically targeting mutated variants of well-known oncogenes in cancer cells [5,6]. It could be safely said that immunotherapies were not on their radar.

Nevertheless, it must be acknowledged that certain immunotherapies were already being given to some patients with limited success: those who did not respond to the standard care of treatments; for example, high-dose interleukin (IL)-2 and interferon (IFN) therapies [7,8,9,10,11], T cell transfer of tumor-infiltrating T cell (TIL) clones expanded ex vivo, or even autologous T cells genetically modified to express recombinant T cell receptors (TCRs) specific for well-known tumor-associated antigens [12,13,14,15].

Everything started to change when an anti-cytotoxic T-lymphocyte-associated protein (CTLA)-4 antibody produced significant therapeutic efficacies in metastatic melanoma patients [16,17]. However, the revolution came in 2012 after the publication of clinical trials evaluating anti-PD-1 and anti-PD-L1 antibodies in cancer patients [18,19]. PD-1 (cluster of differentiation 279, CD279) was discovered in 1992 in apoptotic T-cells by the research team led by Tasuku Honjo [20]. A few years later, PD-L1 was discovered by the team led by Lieping Chen and was named B7 homologue-1 (B7-H1, CD274), a novel member of the B7 family of immunomodulators [21]. PD-L1 turned out to be a negative regulator of immune responses [22,23,24]. Shortly after its discovery, B7-H1 was identified as the canonical ligand of PD-1 and renamed as PD-L1 [24,25]. Indeed, the PD-1/PD-L1 signaling axis was found to regulate inflammation and systemic tolerance [26,27,28,29,30,31,32]. However, most importantly, this signaling axis was critical for the evasion of cancer cells from the immune system [23,33,34,35]. Disruption of PD-1/PD-L1 interactions through several approaches, such as blockade with monoclonal antibodies targeting either PD-1 or PD-L1, led to an enhancement of anti-cancer immune responses in several tumor models [36,37,38,39,40].

Our team began studying PD-1/PD-L1 signaling in 2005, when these molecules were among many novel potential regulators of anti-cancer immune responses. In line with studies conducted by our contemporaries, we found that silencing PD-L1 in antigen-presenting dendritic cells (DC) notably enhanced anti-tumor immunity, particularly when combined with DC activators [31,41]. In 2012, there was a turning point that took everybody by surprise. The oncology field was shaken after the publication of the first clinical trials with PD-1/PD-L1 blocking antibodies [18,19]. The results were truly groundbreaking, demonstrating good tolerability and remarkable therapeutic responses across a wide range of cancers. These outcomes exceeded the therapeutic efficacy previously observed with ipilimumab, a clinically approved anti-CTLA-4 antibody [17]. These results validated the therapeutic potential of treatments that focus on targeting the immune cell compartment rather than the cancer cell itself. Within a few years, medical oncologists had to adapt their expertise to include immunology, and pharmaceutical companies shifted their investments towards developing antibodies targeting the so-called “immune checkpoints” [42]. Immunooncology, as we understand it today, had been born.

## 2. Programmed Death 1 Ligand 1, PD-L1

**Figure 1:**
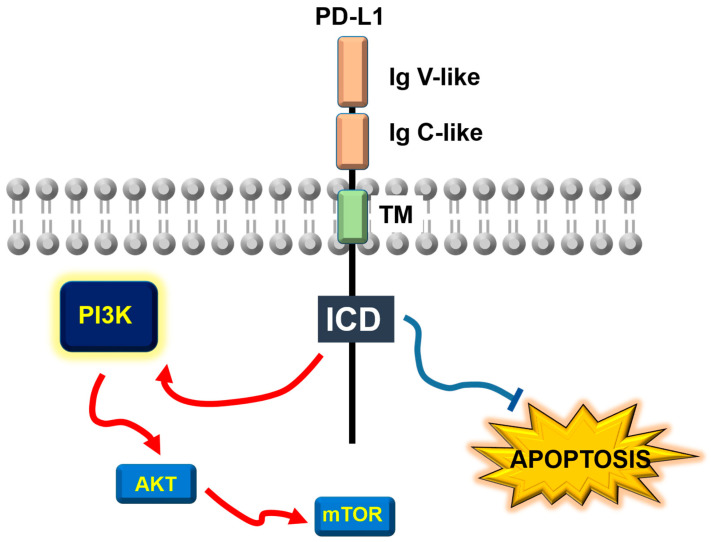
Overall structure and signal transduction functions of programmed death-1 ligand 1. Schematic representation of the molecular organization of PD-L1 in the cell membrane. The reverse signaling activating the PI3K/AKT/mTOR pathway and counteracting apoptosis are indicated. The blue line shows inhibitory relationship between PD-L1 reverse signaling and induction of apoptotic pathways. Red arrows show the activating relationships between the indicated proteins. Ig V and Ig C indicate immunoglobulin-like variable and conserved sequence motifs. TM, transmembrane domain; ICD, intracellular cytoplasmic domain; PI3K, phosphatidylinositol 3-kinase; AKT, AKR mouse T cell lymphoma; and mTOR, molecular target of rapamycin.

PD-L1 is a transmembrane molecule belonging to the B7 family of co-stimulatory molecules. As such, its domain organization is typical to that of the B7 family [21] (Figure 1). PD-L1 is a type I transmembrane surface glycoprotein expressed constitutively by most cells of the myeloid lineage, although its expression can be induced in many cell types following responses to inflammatory stimuli. In cancer cells, PD-L1 expression can also be driven by some oncogenes. PD-L1 presents an immunoglobulin (Ig) variable (V)-like extracellular region, which contains complementary determining-like (CDR) regions constituting the PD-1 binding domain. This interaction is structurally similar to the binding of antigens to antibodies and to the TCR [43,44]. The hydrophobic transmembrane domain is followed by an intracellular region which shows poor sequence similarity to the domains of other B7 counterparts [45]. There is recent evidence suggesting that PD-L1 forms dimers and tetramers in the cell membrane through association by residues in the transmembrane and intracellular domains [46]. Most importantly, its intracellular domain plays a crucial role in reverse signaling across various cell types, including cancer cells, and in maintaining the molecule’s molecular stability.

PD-L1 is expressed constitutively in myeloid cells such as DCs, myeloid-derived suppressor cells (MDSCs), and macrophages [29,31,47,48,49,50,51,52]. Additionally, PD-L1 expression is upregulated in many cell types of different ontologies such as cancer cells, endothelial cells, and T cells [27,53,54,55]. PD-L1 expression can be induced following stimulation with pro-inflammatory cytokines such as interferon gamma (IFNγ), IFN alpha (IFNα), tumor necrosis factor alpha (TNFα), and IL-1 or IL-6 through direct or indirect pathways [56,57,58,59,60]. In cancer cells, transcriptional regulation of PD-L1 expression can differ depending on the tumor type or mutational profile in tumor cells [61,62,63]. In certain cases, cancer cells may constitutively express PD-L1 as a result of the oncogenic activation of signaling pathways, such as those regulated by rat sarcoma (RAS), epidermal growth factor receptor (EGFR), and mitogen-activated protein kinases (MAPK), among others [57,64,65,66,67,68,69]. PD-L1 expression is also regulated by various mechanisms, including the control of its stability and membrane trafficking through processes such as ubiquitination, glycosylation, other protein modifications, and epigenetic regulation [68,70,71,72,73,74,75,76,77,78,79].

## 3. PD-L1 as an Anti-Apoptotic Receptor and AKR Mouse T Cell Lymphoma/Molecular Target of Rapamycin (AKT/mTOR) Regulator

Just before 2012, most of the researchers in PD-1/PD-L1 signaling were focusing on the effects of PD-L1 binding to PD-1 and in the signaling pathways regulated by PD-1 that inhibited T-cells. Thus, among these where the pathways associated to the binding of Src homology region 2 domain-containing phosphatases (SHP) 1 and 2 to immunoreceptor tyrosine-based inhibitory (ITIM) and immunoreceptor tyrosine-based switch (ITSM) motifs in the intracellular domain of PD-1 [80,81,82,83,84,85,86,87], and the up-regulation of E3 ubiquitin ligases of the Casitas B cell lymphoma (CBL) family [31,88,89,90]. At that time, little attention was given to the possibility of reverse signaling by PD-L1 within cancer cells themselves, with the exception of a few pioneering studies led by Lieping Chen’s team. For several years, the protective role of PD-L1 was understood primarily as its function as a molecular brake, inhibiting the effector activities of cancer-specific T cells through its interaction with PD-1. In this model, PD-1 signaling within the T-cell was considered the primary mechanism by which tumors defended themselves against T-cell attacks. In 2004, a screening for T-cell inhibitory molecules expressed by melanoma B16-F10 cancer cells treated with IFNγ uncovered PD-L1 as a protective candidate. As expected, antibody blockade of PD-L1/PD-1 interactions, or absence of PD-1 expression in T cells led augmented tumor rejection, suggesting that PD-L1 represented a molecular shield protecting cancer cells from T-cell cytotoxicity [34,35,36]. However, and surprisingly, in 2008, Lieping Chen’s group provided a molecular protection mechanism for cancer cells driven by PD-L1 independently on its PD-1-engaging activities. In this model, PD-L1 expression conferred cancer cells with resistance to different apoptotic stimuli by transmitting intracellular signals to cancer cells [91] (Figure 1). The authors of the study demonstrated that P815 mastocytoma and renal adenocarcinoma Renca cell lines required PD-L1 expression to resist T cell cytotoxicity. As expected, this resistance was abrogated by PD-L1 blocking antibodies. Then, the authors used T cells expressing a PD-1 molecule with its intracellular region replaced by GFP, rendering a PD-1 signal-null. In these conditions, cancer cells remained resistant to the T cell attack as long as they expressed PD-L1. This protection was lost when cancer cells expressed a PD-L1 molecule with its intracellular domain replaced by GFP. This study concluded that PD-L1 intracellular signaling conferred resistance to cancer cells against pro-apoptotic stimuli, including first apoptosis signal receptor (Fas)-Fas ligand (FasL) interactions, among others [91]. However, when the authors examined major anti-apoptotic and apoptotic pathways, they did not find significant differences between controls versus PD-L1-engaged cancer cells. This study concluded that PD-L1 may be providing an early signal to inhibit apoptotic pathways. Interestingly, the potential signal transduction properties of PD-L1 were described in other experimental contexts. One of such contexts was metabolic competition between cancer cells and T cells. This mechanism is utilized by cancer cells by depleting glucose in the tumor microenvironment. Glucose depletion then interferes with T cell effector activities [92]. A study conducted by Palmer et al. in 2015 demonstrated that treatments with antibodies targeting immune checkpoints CTLA-4, PD-1, and PD-L1 restored glucose concentrations within the tumor microenvironment [93]. These findings indicated that immune checkpoint molecules played a role in regulating glucose metabolism in mouse sarcoma cells. The authors further investigated the impact of a PD-L1 blockade or silencing on glucose metabolism and demonstrated that a PD-L1 blockade, even in the absence of T cells, inhibited the AKT/mTOR pathway (Figure 1). This inhibition led to a reduction in transcription of glycolytic genes, decreasing glucose consumption without affecting cancer cell proliferation or tumor growth. This direct effect of PD-L1 expression over glucose metabolism was later confirmed in 2019 by Kim et al. in lung cancer cell lines [94]. Regulation of mTOR and autophagy pathways by PD-L1 expression in the absence of T cells was also demonstrated by Clark et al. in 2016 in two cancer models: mouse B16 melanoma and ovarian ID8 cells [95]. In this case, however, the reduction in PD-L1 expression inhibited cancer cell proliferation, indicating that these effects may be cell type-specific, as similar results were also reported in U87 glioblastoma cells [96]. Moreover, the same authors concluded in a follow-up study that PD-L1 signaling played a role in tumor-initiating cells (TIC) and their resistance to pro-apoptotic treatments. PD-L1 silencing reduced TIC numbers and their associated functions. However, unlike the findings by Chen’s team and our own results [91,97], PD-L1 expression in TIC sensitized these cells to IFNγ and rapamycin [98].

A wide array of subsequent studies confirmed the activating role of PD-L1 intrinsic signaling in the PI3K-AKT-mTOR pathways typically linked to enhancing cancer cell survival and resistance to apoptosis [99,100,101,102] (Figure 1).

## 4. PD-L1 Non-Classical Signaling Motifs and Resistance to Interferons in Immunotherapy

In 2017, the research team led by Antoni Ribas published a groundbreaking study demonstrating a mechanism by which some melanoma tumors become resistant to PD-1 blockade therapies [103]. The study’s authors conducted whole-exome sequencing on biopsies from patients with advanced melanoma undergoing PD-1 blockade monotherapy, both before and after treatment. Loss-of-function mutations in proteins within the Janus kinase (JAK)1/JAK2 signaling pathways and antigen presentation processes were identified in non-responders, rendering cancer cells unresponsive to interferons (IFNs). The authors concluded that resistance to immunotherapy stemmed from the suppression of PD-L1 upregulation in response to IFN stimulation. Reduced surface expression of PD-L1 was linked to unresponsiveness to PD-1 blockade due to the absence of PD-L1/PD-1 interactions. These findings suggested a strong connection between the inactivation of IFN signaling in cancer cells and resistance to PD-1/PD-L1 blockade immunotherapy [104]. These observations were recently widened to include mutations of mediators of the stimulator of interferon genes (STING) pathway [105]. It could be argued, however, that the absence of PD-L1 expression in cancer cells should also remove the inhibitory effect on T-cells, regardless of whether they express PD-1. Consequently, T-cells could theoretically “be free” to exert cytotoxic effects on PD-L1-negative cancer cells. This raised the need for additional resistance mechanisms to explain this apparent paradox.

**Figure 2:**
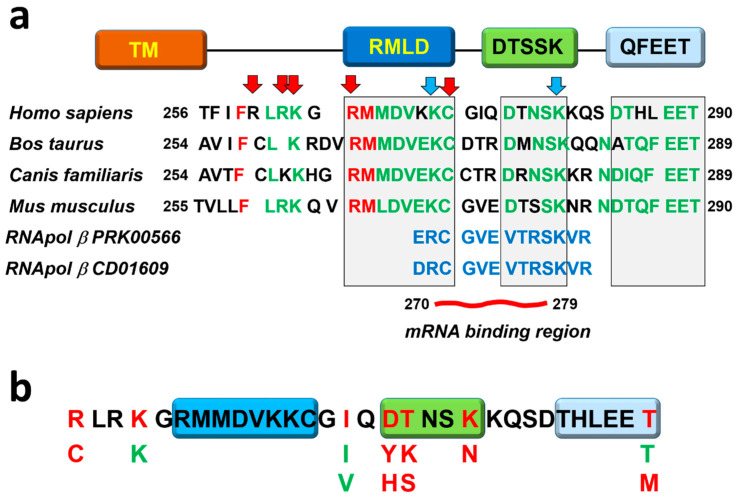
Conserved motifs in the cytoplasmic domain of programmed death-1 ligand 1. (**a**) On top, the transmembrane and intracellular region of PD-L1, with the conserved signaling motifs RMLD(VEKC), DTSSK, and QFEET highlighted in boxes. Below, multialignment of selected PD-L1 sequences from the species indicated on the left, or with the indicated bacterial DNA-dependent RNA polymerase β subunit conserved motifs. Red arrows show arginine and lysine residues involved in PD-L1 stability; blue arrows, show lysine residues with IFN-regulating activities. Residues in red show highly conserved aminoacids; residues in green show conserved aminoacids; the region binding mRNAs is indicated; and numbers indicate aminoacid positions. (**b**) Selection of mutations in PD-L1 found in human carcinomas. On top is the human PD-L1 sequence including the homologous RMLD(VEKC), DTSSK, and QFEET motifs. Below, mutations found in biopsies from human patients. In red, disruptive mutations; in green, conserved mutations are shown. RNApol, RNA polymerase.

The molecular connection between PD-L1 signal transduction and resistance to IFN-induced apoptosis was uncovered through a fortuitous observation by our team. We were developing living cell-based melanoma vaccines aimed at enhancing their immunogenicity by combining IFNβ secretion with PD-L1 silencing, using an approach that had previously proven effective for other cytokines [41]. While living melanoma cell lines overproducing IFNβ or with silenced PD-L1 could be derived, it was impossible to keep living melanoma cells with both features [97]. We decided to pursue why this was the case, and found that cells died from IFN-induced apoptosis when PD-L1 expression was reduced. We proved that the critical feature for survival of melanoma cells was the expression of PD-L1. Its silencing, deletion, or blockade with antibodies caused the death of melanoma cells expressing IFNβ. Moreover, the protective activities of PD-L1 were eliminated by truncation of its intracellular region. All results pointed to PD-L1 possessing intracellular signaling capacities that interfered with IFN-driven apoptosis. Although the research team led by Lieping Chen explored this issue and identified no specific pathways altered by PD-L1, we adopted a traditional approach focused directly on PD-L1 itself [97]. First, we performed multicomparisons of sequences from mammalian PD-L1 molecules. Interestingly, three sequence motifs showed high phylogenetic conservation, “RMLDVEKC”, “DTSSK”, and “QFEET”, as present in the murine PD-L1 sequence (Figure 2). A surprising result from our original bioinformatic analyses on PD-L1 was the identification of a conserved sequence in the intracytoplasmic domain corresponding to a highly conserved bacterial DNA-dependent RNA polymerase β subunit motif (Figure 2b) [45,97]. This result was completely unexpected. The RNApol motif encompassed part of the RMLD and the complete DTSSK sequences (Figure 2b). We demonstrated that these sequences contributed to the anti-IFN response, but the relevance of an RNApol motif in PD-L1’s carboxy terminus was unclear at the time. However, in 2019, Tu et al. demonstrated that PD-L1 could act as an RNA-binding protein [106], mapping the interacting domain (aminoacids 270–279) to most of the RNApol-like motif (Figure 2b). The authors of this study showed that intracellular PD-L1 stabilized several mRNAs, including those coding for nibrin (NBS1) and BRCA1 in HCT116 and MDA-MD-231 cell lines. PD-L1 protected mRNAs from degradation and increased resistance to DNA damage. It is tempting to speculate that the RNApol-like motif contributes to DNA/RNA binding and participates in transcriptional regulation and DNA repair [70,71,107,108,109,110,111,112].

**Figure 3:**
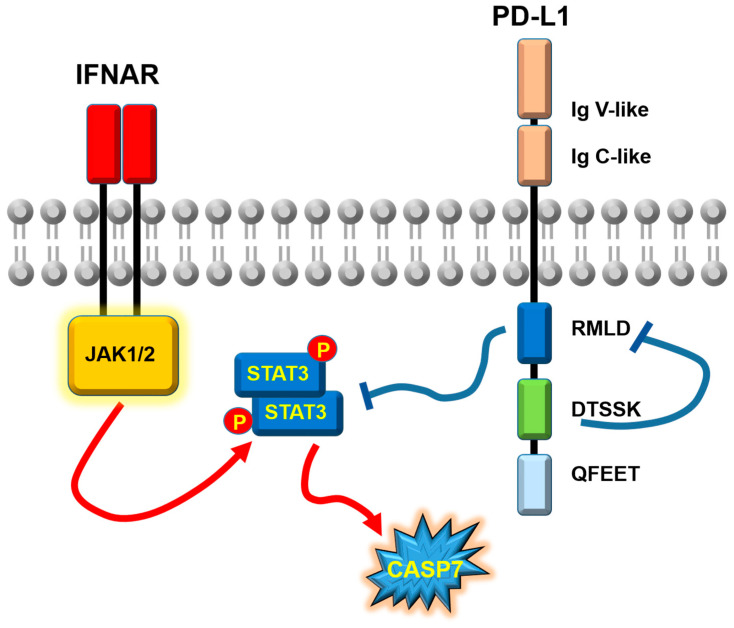
Structure, conserved motifs, and anti-interferon activities of programmed death-1 ligand 1. Schematic representation of the molecular organization of PD-L1 (**right**) in the cell membrane, with the main conserved sequence motifs indicated in the figure. The reverse signaling pathway counteracting IFN-driven apoptosis is also depicted. On the (**left**), IFNs signal through JAK1/2 leading to STAT3 phosphorylation and CASP7-dependent apoptosis. Blue lines show inhibitory relationships between the indicated sequence motifs and molecules. Red arrows show activating relationships between the indicated proteins. Ig V and Ig C indicate the immunoglobulin-like variable and conserved sequence motifs. IFNAR, interferon receptor.

Using a classical deletion approach, the “RMLDVEKC” motif was demonstrated to be absolutely required to inhibit type I and II IFN-driven apoptosis (Figure 3). In contrast, the removal of the “DTSSK” motif enhanced the anti-IFN activities of PD-L1, highlighting its role as a negative regulator for this specific motif. The expression of cell surface proteins is often regulated by ubiquitination. To investigate this further, the lysine residues in the “RMLDVEKC” and “DTSSK” motifs were mutated to rule out this possibility. These mutations also enhanced the anti-IFN activities of PD-L1, further supporting the idea that these lysine residues acted as negative regulators [97], without apparent changes in PD-L1 cell surface expression were observed in our study. Interestingly, later studies demonstrated a key role for residue modifications in the carboxy-terminus of PD-L1 that regulate its stability and degradation. For example, arginines R260, R262, and R265, just upstream of the RMLDVEKC motif, were shown to be critical for PD-L1 dimerization and for its complex glycosylation (Figure 2a) [46]. Acetylation of K263 regulated its nuclear localization [113,114]. Additionally, R260 and K263 were critical for huntingtin interacting protein 1 related (HIP1R) binding, which targeted PD-L1 for degradation [115]. Interestingly, we previously found mutations within some residues of these motifs selected in skin cancers and other carcinomas [97], highlighting their critical role in carcinogenesis (Figure 2b). Other modifications in the carboxy-terminus regulate PD-L1 stabilization. For example, palmitoylation in C272 by zinc finger DHHC-type containing 3 (DHHC3) stabilizes PD-L1, permitting cancer cells to escape from the T cell attack [116]. This palmitoylation inhibits ubiquitination of C272 that causes PD-L1 degradation [117]. Surprisingly, we did not find specific functions for the “QFEET” motif [97].

Overall, IFN signaling pathways constitute a major mechanism for response to PD-1/PD-L1 blockade [45,90,97,118,119,120]. Hence, it is no wonder that one of the key functions of PD-L1 in cancer cells is to counteract IFN signaling and reduce IFN-induced apoptosis, although the specific pathway may be cancer type-dependent [121]. To explore a potential mechanism, we examined IFN signal transduction and the caspase-dependent regulation of apoptosis in melanoma cells [97] (Figure 3). Melanoma cells responded to IFNβ stimulation by enhancing STAT3 upregulation, but not STAT1 or STAT2. The elimination of PD-L1 selectively induced signal transducer and activator of transcription (STAT)3 phosphorylation in tyrosine 705, but not serine 727 phosphorylation. Abrogation of PD-L1 expression in melanoma cells sensitized them to IFN-driven apoptosis through a pathway dependent on caspases 7 and 9. Overall, our results indicated that the “RMLDVEKC” motif, negatively regulated by the “DTSSK” motif, inhibited STAT3 Y705 phosphorylation and IFN receptor signaling, truncating apoptosis dependent on caspase 7 and 9. Indeed, PD-L1 abrogation in macrophages was shown to potentiate STAT3 activation leading to IL6 production [122]. Something similar was observed in MDSCs [123]. Interestingly, a different mechanism involving phospho-STAT3 was described whereby Y705-STAT3 physically associates with PD-L1, facilitating its nuclear transport and pyroptosis induction in cancer cells through caspase 8 [108]. In other tumor models, the relationship between PD-L1 expression and regulation of JAK-STAT signaling pathways may differ. For example, JAK-STAT-activation has been shown to occur in NSCLC cells caused by PD-L1 reverse signaling in the context of enhancement of angiogenesis, as recently shown by Cavazzoni et el [124].

Melanoma B16-derived tumors were more sensitive to IFNβ when PD-L1 expression was reduced, a feature dependent on PD-L1 intracellular signal transduction in B16 cells. Indeed, we also found that the human homologue of the “DTSSK” motif was selectively enriched in mutations in several human carcinomas (Figure 2b). These mutations were tested in vitro, demonstrating that they enhanced the anti-IFN capacities of human PD-L1 [97]. Importantly, an antibody blockade of PD-L1 decreased cancer cell proliferation and enhanced sensitivity to IFNβ in murine and human melanoma cancer cells [97], a mechanism later confirmed in other tumor types [125,126]. The role of PD-L1 as a factor favoring cancer cell proliferation was also supported by studies in other tumor models, in which agonistic PD-L1 antibodies induce cell growth and survival, such as in Hodgkin lymphoma [127]. Thus, apart from inactivating mutations of the IFN signal transduction pathway and antigen presentation, other mutations directly in PD-L1 can be selected in human carcinomas that protect cancer cells from IFN-induced apoptosis.

## 5. The PD-L1 Interactome

**Figure 4:**
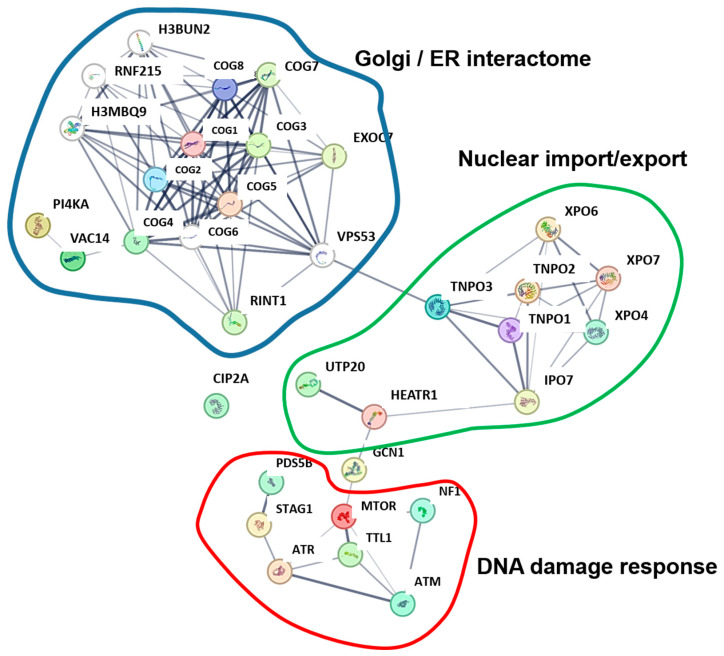
Interactome associated to human PD-L1. Interactors detected experimentally in [128] are shown and arranged reconstructing their interactions with STRING (https://string-db.org/, accessed on 1 March 2025) [45]. Predicted nodes are shown in white. Interactomes have been grouped in three main classes according to their associated functions, as indicated in the figure.

We continued with a follow-up study taking advantage of the fact that the human interactome had been published [128]. These data were obtained by mass spectrometry analyses in human embryonic kidney (HEK)-293T cells after antibody-based affinity purification of targets of interest bound to their interacting proteins. An interactome associated to human PD-L1 was constructed for the first time with proteins associated to affinity-purified PD-L1 [45]. This interactome can be divided in three functional groups: (1) DNA-damage response; (2) nuclear protein and RNA import/export group; (3) and Golgi-endoplasmic reticulum (ER) interactors (Figure 4).

The first group of proteins included mTOR, several kinases that regulate ancho-independent cell growth, and surprisingly, key regulators of the DNA damage response pathway including ataxia telangiectasia mutated (ATM) and ataxia telangiectasia and Rad3-related protein (ATR) (Figure 4) [45]. It had already been experimentally demonstrated at the time that PD-L1 was regulating signaling through mTOR/AKT pathways [100], as extensively described above. However, the association of ATM and ATR proteins to PD-L1 was unexpected, strongly suggesting a direct link between PD-L1 and regulation of the DNA damage response pathway [45]. Indeed, radiotherapy had been found to induce PD-L1 upregulation in cancer cells through the activity of ATM/ATR [129]. Later on in 2021, Ozawa et al. showed that IFN-mediated PD-L1 upregulation was associated to the double-strand breaks (DSB) repair pathway in patients with colitic cancer, mainly with immunofluorescence microscopy techniques [130]. Indeed, PD-L1 upregulation was shown to be associated with resistance of cancer cells to radiotherapy in a genome-wide CRISPR/Cas9-based screening by accelerating DBS repair. Interestingly, this mechanism was dependent on PD-L1 N219 deglycosylation and its translocation to the cell nucleus. PD-L1 translocation was mediated by CMTM6, one of PD-L1’s known interactors [70,71] (Table 1). In the nucleus, PD-L1 was shown to bind KU (X-ray repair cross complementing 6 and 5 dimer, XRCC6/XRCC5) through its IgC-like domain and facilitate non-homologous end-joining recombination (NHEJ) [112]. Further research supported that nuclear PD-L1 was not likely an artefact as suggested by other authors [131], and confirmed that PD-L1 can translocate to the nucleus [107,112,114,132]. Hou et al. showed in a study that PD-L1 translocated to the nucleus through an interaction of its carboxy terminus with Y705-phosphorylated STAT3, where PD-L1/STAT3 regulated gasdermin C (GSDMC) transcription in cancer cells in response to hypoxia [108] (Table 1). Similar links between PD-L1 and DNA repair mechanisms have been also published by other authors [109,110]. Very recently, a direct association between nuclear PD-L1 and ATR, which regulates DNA damage response, was demonstrated [111] (Table 1). A role for regulation of DNA repair mechanisms through binding to BRCA1-associated RING domain 1 (BARD1) was also described in several cancer types [109]. The authors of this study suggested that PD-L1 may be promoting BARD1 translocation into the nucleus, favoring breast cancer 1 (BRCA1) nuclear foci formation and homologous recombination. However, the authors of this study did not map the potential binding site for BARD1.

The second group of interactors that we originally described was associated to mRNA import/export and protein nuclear import/export pathways (Figure 4) [45]. At that time, we did not consider these results to be relevant, but since then, many research teams demonstrated the nuclear import/export of PD-L1 associated to glycosylation and deglycosylation pathways, binding to phosphorylated STAT-3, CKLF such as MARVEL transmembrane domain containing 6 (CMTM6) binding, or by other mechanisms (Table 1). As mentioned above, it turned out that nuclear PD-L1 is related to key and highly relevant functions in cancer cell biology, inflammation, and immunity [55,107,109,111,112,114,130,132].

The third group of interactors included Golgi and ER proteins, as would be expected for a transmembrane glycoprotein. Indeed, the regulation of PD-L1 transport, ubiquitination, and degradation turned out to be a major immunoregulatory mechanism in tumor biology [31,70,71,72,74,75,76,77,78,79,112,132,133,134,135].

In 2021, a PD-L1-associated metabolic interactome was also obtained and its relationship with choline kinase (CHK)α was established by analyzing breast cancer (MDA-MB-231 and SUM-149) and pancreatic ductal adenocarcinoma (Pa09C and Pa20C) cell lines [136]. The authors used high-resolution magnetic resonance spectroscopy, following PD-L1 and CHKα silencing with small-interfering RNAs. An inverse correlation in expression between both proteins was demonstrated. Inverse changes in metabolites were identified and regulated by both proteins, as well as lipid profiles, which were mediated through CHKα, cyclooxygenase 2 (COX2), and transforming growth factor beta (TGFβ).

A later study by Nieto et al. defined PD-L1’s intracellular interactome and the role of its carboxy-terminus in mediating signal transduction in a head and neck squamous cell carcinoma model [137]. The authors used humanized head and neck squamous cell carcinoma (HNSCC) in vivo models bearing also T-cells. Therefore, while PD-L1 blockade alone was not sufficient to achieve tumor growth control, its combination with STAT3 inhibition achieved significant therapeutic activities. Interestingly, there were some differences between the interactome in the absence and presence of PD-1 binding to PD-L1. The authors of the study also demonstrated a physical association between PD-L1, interleukin enhancer binding factor (ILF)2, ILF3, family with sequence similarity 129 member B (FAM129B), and protein tyrosine phosphatase non-receptor type 1 (PTPN1) by co-immunoprecipitation (Table 1). Interestingly, ILF2 and ILF3 associated with PD-L1 in the absence and in the presence of PD-1 interaction. In this experimental model, ILF2-ILF3 binding to PD-L1 preceded and induced STAT3 phosphorylation, favoring cancer cell progression.

Other direct interactors identified to date include glycogen synthase kinase 3 beta (GSK3β) in head and neck squamous carcinoma [138] (Table 1). Upregulated PD-L1 following irradiation in radioresistant cancer cells enhanced its association with GSK3β, leading to its inactivation by phosphorylation and subsequent increase in cell proliferation and survival. AKT was also found to be a direct interactor at least by coimmunoprecipitation with PD-L1 in platelets, sustaining its activation [101] (Table 1).

In triple-negative breast cancer cells, PD-L1 was shown to co-immunoprecipitate with the tyrosine phosphatase PTP1B through PD-L1’s carboxy terminus (Table 1). However, the specific motifs mediating this binding were not identified in this study. The authors of the study proposed a mechanism by which PD-L1 binding to PTPB1B prevented GSK3β-mediated phosphorylation and subsequent degradation by ubiquitination of Snail, promoting epithelial to mesenchymal transition (EMT) in cancer cells [139].

The deubiquitinating enzyme OTU deubiquitinase ubiquitin aldehyde binding 2 (OTUB2) was also shown to be a direct interactor with cytoplasmic PD-L1 in many cancer cell types [140]. Interestingly, this interactor, at least its catalytically active form, was shown to bind to PD-L1’s intracellular domain both in vivo and also in vitro with purified proteins (Table 1). However, the specific motif mediating this interaction was not mapped. The authors of this study proposed that catalytically active OTUB2 binds to PD-L1 and prevents its degradation.

Intriguingly, karyopherin subunit beta 1 (KPNB1) has been shown to associate to PD-L1 and mediate its translocation to the nucleus, where it cooperated with specificity protein 1 (SP1) to transactivate growth arrest specific 6 (GAS6) transcription, leading to enhanced non-small cell lung cancer (NSCLC) proliferation [107] (Table 1). Likewise, nuclear PD-L1 has been shown to associate with the epidermal growth factor receptor 1 (EGR1) promoter, leading to its transcription by association with phospho-STAT3 [114]. These results extend PD-L1’s role to that of transcriptional co-activator, if not a transcription factor by itself.

Since the early publications on PD-L1 intrinsic signaling in cancer cells, subsequent research reinforced its critical role in a plethora of biological regulatory mechanisms associated with its binding to a collection of different interactors. Indeed, the combination of anti-PD-L1 antibody with IFN treatment of BRAFV600E PTEN-/- melanoma cells was shown to activate NOD-like receptor protein 3 (NLRP3) through protein kinase R (PKR) binding to regulate the inflammasome (Table 1). This effect was shown to take place in LLC mouse lung adenocarcinoma cells. This mechanism was driven by PD-L1 crosslinking and STAT3 suppression, which seems to be a central common mechanism in PD-L1 intrinsic signaling in several cancer cell models [97,141].

PD-L1 has been shown to directly bind phospholipase C in an EGFR-dependent manner in several lung cancer cell variants, regulating Rho GTPases, which enhanced tumorigenicity [126]. PD-L1 interactors were probed in a yeast two-hybrid assay, and filamin A (FLNA) and phospholipase C gamma 1 (PLCγ1) were found. The authors of this study concluded that PD-L1 associated with PLCγ1, which enhanced its transphosphorylation by EGFRs, and mapped the interacting domain at the region containing the DTSSK motif (Table 1). Binding of PD-L1 to H-RAS was shown by co-immunoprecipitation in 293T and LN229 cells by Qiu et al., although the specific domain regulating this interaction was not described in their study [142] (Table 1). The authors showed that PD-L1 promoted RAS activation, leading to EMT in glioblastoma multiforme. A similar mechanism was observed in colorectal cancer cells, regulated through the ERK/AKT signal transduction pathway after binding to K-RAS, as shown by co-immunoprecipitation [102]. In this study, PD-L1/PD-L1 interactions mediated extracellular signal-mediated kinase (ERK)/AKT activation, although the specific domain of PD-L1 associated to K-RAS was not identified (Table 1). EMT was recently shown to be induced by PD-L1 intrinsic signaling, but in this study, a MAPK p38-dependent mechanism was involved [143]. Hence, PD-L1 expression activated the MAPK ERK pathway, accelerating cancer cell growth and tumor progression.

## 6. Reverse Signaling of PD-L1 in Immune Cells

Intrinsic PD-L1 reverse signaling is not restricted to cancer cells or exclusively linked to immune escape. PD-L1 reverse signaling has been demonstrated both in CD4 and in CD8 T cells [144,145]. In a study by Diskin et al., binding of PD-L1 induced STAT3-dependent reverse signaling in CD4 T cells. This back-signaling mechanism prevented activation, but also enhanced Th17 differentiation in detriment to Th1 polarization. The same authors also tested PD-L1 reverse signaling in CD8 T cells, which became anergic by acquiring a t-bet-negative, IFN-γ-negative phenotype. Interestingly, Fanelli et al. demonstrated that inducing PD-L1 reverse signaling in memory CD4 T cells led to their differentiation into regulatory CD4 T cells. This study was carried out in the context of TCR stimulation through inhibition of TCR-dependent ERK activation.

PD-L1 on dendritic cells limits autoimmunity and follicular T cell differentiation and function [146]. Theivanthiran et al. highlighted the role played by PD-L1 associated with the inflammasome to drive granulocytic MDSC recruitment into the tumor, which caused resistance to PD-1 blockade immunotherapy [141]. Lucas et al. demonstrated a critical role for PD-L1 reverse signaling in regulating the migration of conventional activated DCs from the skin to the draining lymph node [147]. This mechanism was shown to be required for proper T cell priming, and relied on activation of c-c chemokine receptor type 7 (CCR7) signaling through the DTSSK domain of PD-L1. Most importantly, the DTSSK domain in PD-L1 was found to also be critical in lymphatic endothelial cells (LECs) for lymphatic remodeling following immune stimulation with polyIC, TNFα, and IFNβ [148]. Inactivating mutations in the DTSSK domain by replacing the TSS motif with alanines impeded actin polymerization and LEC movement. Interestingly, these mutations reduced the binding of PD-L1 to paxillin and phosphorylated S727 and Y705 STAT3 required for protein assembly at focal adhesions (Table 1). In this study, the authors further characterized PD-L1 interactors and phospho-interactors after immunoprecipitation, finding five differential proteins between wild-type PD-L1 and DTSSK-mutated PD-L1 [148].

## 7. Conclusions and Further Research

The clinical use of PD-L1/PD-1 blockade immunotherapies came fast and developed faster. Much faster than research into the molecular and cellular mechanisms of PD-1 and PD-L1 functions. Nevertheless, the extraordinary success of these therapies ignited research into the functions of these immune checkpoint inhibitors. During the last 10 years, we discovered multiple facets of PD-L1 in cell biology. Not only as the so-called T-cell break in immunotherapy, but also as a molecule actively participating in cell proliferation, survival, and in DNA damage responses. The importance of nuclear PD-L1 has also arisen recently, suggesting that PD-L1 can function as a co-transcriptional activator as well.

In our opinion, PD-L1 may possess additional functions related to its transcriptional activation capacities. Although research on this aspect remains limited, one study suggested that PD-L1 functions as an RNA-binding protein. However, this property requires further investigation and confirmation through independent studies.

Expanding upon this idea, it is tempting to speculate that PD-L1 may also exhibit DNA-binding properties, potentially facilitating its interaction with cellular promoters and/or enhancers. This possibility could open new avenues for understanding its role in gene regulation. Along this line of thought, exploring the relationship between its carboxy-terminal region and domains from RNA polymerases could provide valuable insights into its function. Investigating whether PD-L1 directly associates with components of the transcriptional machinery may reveal previously unknown regulatory mechanisms.

Furthermore, identifying a broader array of molecular interactors—including kinases and transcription factors—could expand therapeutic strategies targeting PD-L1. This knowledge may allow for the development of combination treatments that incorporate small-molecule inhibitors aimed at disrupting its interactions. Given the evolving landscape of oncology, researchers and clinicians are increasingly focusing on optimizing combinatorial approaches that integrate chemotherapy and targeted therapies with PD-1 and PD-L1 blockade strategies. These efforts aim to enhance treatment efficacy, minimize resistance, and improve patient outcomes.

It has to be remarked that this review focused on the structure and functions of the canonical PD-L1 molecule. Nevertheless, several types of PD-L1 isoforms can be generated by alternative splicing that can both play additional roles and alter the efficacy of PD-1/PD-L1 blockade strategies. Several variants have been described, including those lacking the Ig variable domain (PD-L1Δ3) [149] and many secreted spliced variants as extensively reviewed in [150]. Apart from these classical spliced isoforms, it is worth mentioning the description of a long non-coding RNA isoform of PD-L1 generated by alternative splicing, without correlation with the actual expression of PD-L1 in lung adenocarcinoma cells [151]. This RNA, termed PD-L1-Inc, increases proliferation and inhibits cancer cell apoptosis through activation of c-Myc. Indeed, the existence of multiple PD-L1 isoforms complicates the landscape of tumor immunology. For example, isoforms such as PD-L1-Inc actively promote aggressive tumor phenotypes, while protein variants such as PD-L1Δ3 may affect how tumors escape immune surveillance. The expression of these isoforms can affect the response to immunotherapies but can also alter decision-making processes in treatment courses: Since PD-L1 expression is commonly used as a biomarker to select candidates for checkpoint blockade therapies, the diverse nature of these isoforms could lead to discrepancies in patient classification. Nevertheless, it is also our opinion that a refined understanding of these isoforms opens up new therapeutic avenues. Combining the conventional PD-L1 blockade with strategies that target specific splice variants or counteract their downstream effects might enhance overall treatment efficacy.

The knowledge acquired of its structural organization, functions, and interactors will help us in the identification of novel targets and in designing improved therapies that can be used in combination with standard immune checkpoint blockade immunotherapies.

## Figures and Tables

**Table 1:** Known interactors of PD-L1.

Name	Role	Interacting Site in PD-L1
CMTM6	Nuclear translocation	Transmembrane domain
KU STAT3 ATR BARD1 ILF2 ILF3 FAM129B PTPN1 GSK3β AKT PTPB1B OTUB2 KPNB1 PKR Paxillin H-RAS K-RAS FLNA PLCγ1	DNA repair	Ig C-like site
	Nuclear translocation/transcription DNA repair DNA repair Proliferation Proliferation Proliferation Proliferation Radioresistance Radioresistance Epithelial to mesenchymal transition PD-L1 stability Nuclear translocation/transcription Inflammasome regulation DC trafficking Epithelial to mesenchymal transition Epithelial to mesenchymal transition Tumorigenicity Tumorigenicity	DTSSK motif Unknown Unknown Unknown Unknown Unknown Unknown Unknown Unknown Carboxy-terminus Carboxy-terminus Unknown Unknown DTSSK motif Unknown Unknown DTSSK motif DTSSK motif

## Data Availability

No data were generated.
